# ^198^Au grain implantation for early tongue cancer in patients of advanced age or poor performance status

**DOI:** 10.1093/jrr/rrt060

**Published:** 2013-05-17

**Authors:** Yoshiharu Ryu, Hitoshi Shibuya, Keiji Hayashi

**Affiliations:** Department of Radiology, Tokyo Medical and Dental University, 1-5-45 Yushima, Bunkyo-ku, Tokyo 113-8510, Japan

**Keywords:** ^198^Au grain implantation, brachytherapy, early tongue cancer, advanced age, performance status

## Abstract

Brachytherapy using ^198^Au grains is minimally invasive and the only curative treatment for early tongue cancer in patients of advanced age or poor performance status available in our institution. From March 1993 to February 2008, ^198^Au grains were used to treat a group of 96 Stage I–II tongue cancer patients who could not undergo surgery or brachytherapy using ^192^Ir pins because of an advanced age (≥75 years) or poor performance status (≥2). The patients were followed for 3.9 ± 3.3 years, and the cause-specific survival and local control rates were determined. Survival analyses were performed using the Kaplan-Meier method, and univariate and multivariate analyses were performed using the Cox proportional hazard model. The results were compared with those for a group of 193 early tongue-cancer patients who underwent treatment using iridium pins. The 5-year cause-specific survival and local control rates of the ^198^Au grains group were 71% and 68%, respectively, both of which were 16% lower than the corresponding rates for the ^192^Ir pins group. Our study demonstrated that as the last curative treatment available, ^198^Au grain implantation could be used to achieve moderate treatment results for early tongue cancer in patients of advanced age or poor performance status.

## INTRODUCTION

Brachytherapy delivers a high radiation dose to the tumor while producing limited damage to the adjacent normal tissues. Therefore, this treatment modality preserves the shape and functions of the tongue and allows radiotherapy to be reserved for secondary malignancies of the head and neck [1–4]. Low-dose-rate brachytherapy using ^192^Ir hairpins or ^137^Cs needles is widely used for the treatment of early tongue cancer because it allows excellent local control rates that are comparable to those of surgery [1, 5, 6].

^198^Au grains are alternative sources used in our institution, usually prescribed for patients who cannot tolerate surgery, ^192^Ir hairpins, or ^137^Cs needles because of advanced patient age or poor performance status due to the presence of complications such as dementia, diabetes, or apoplexy [7, 8]. ^198^Au grains are small and can be easily applied under local anesthesia without causing severe pain or discomfort during the insertion and treatment [9]. They have a short half-life of 2.7 days, shortening the hospital stay and making subsequent complications less severe [9]. They are permanent implant sources, eliminating any re-traumatization of the tongue during grain extraction.

With the aging of Japan's population, the incidence rates of tongue cancer are also rising [10]. The demand for treatment with ^198^Au grains in elderly patients is also increasing in our institution. Knowing the clinical outcomes and prognostic factors of this treatment are thus important. However, to the best of our knowledge, this topic has not been previously reported. In this study, we retrospectively examined the clinical outcomes of a group of patients treated with ^198^Au grains (Au-group) and compared them with the outcomes of a group of patients treated with ^192^Ir hairpins (Ir-group).

## MATERIALS AND METHODS

From March 1993 to February 2008, a total of 429 patients diagnosed with primary squamous cell carcinoma of the oral tongue received interstitial brachytherapy at Tokyo Medical and Dental University Hospital. Of the 429 patients, 96 were treated with ^198^Au grains because of advanced age (≥75 years), or poor performance status ( ≥ 2 on the ECOG scale) due to complications such as dementia, diabetes, apoplexy, or chronic obstructive pulmonary disease (COPD). ^192^Ir hairpins were applied to 193 patients. The remaining patients were treated using ^137^Cs needles. We retrospectively examined the clinical outcomes of the Au-group and the Ir-group. We compared these two groups because we wanted to compare the result that could be achieved by applying ^198^Au grains to patients who cannot endure invasive therapy with the best possible result achieved by applying ^192^Ir hairpins. We excluded the Cs-group in this study because ^137^Cs needles are no longer available. This study was approved by the research ethics board committee of the university.

The patient characteristics are shown in Table 1. The mean age of the Au-group was 11 years higher than that of the Ir-group, which was a significant difference. All the patients with a performance status >1 were treated with ^198^Au grains. The rate of patients with complications such as dementia, diabetes, apoplexy or COPD was higher in the Au-group than in the Ir-group, although the difference was not significant. All the complications in the Ir-group were controlled by medications, while all the complications in the Au-group were more severe than those in the Ir-group. All the lesions were histologically confirmed as squamous cell carcinoma and were staged as Stage I or II according to the UICC (International Union Against Cancer 2009) TNM classification [11]. The macroscopic appearances of the tumors were categorized as follows: (i) exophytic: tumors exhibiting external growth; (ii) superficial: flat tumors without palpable infiltration; or (iii) infiltrative: tumors penetrating deeply with or without ulceration. No significant differences in the T-factors or macroscopic appearances were observed between the two groups.

**Table 1.** Patient characteristics

	Au-group (*n* = 96)	Ir-group (*n* = 193)	*P*-value
Age (yrs)	71 ± 13	60 ± 14	<0.0001***
Sex (male/female)	58/38	134/59	0.15
Male (%)	60	69	
Female (%)	40	31	
T-factor (T1/T2)	43/53	66/127	0.09
T1	45	34	
T2	55	66	
Complications (%)	19	11	0.10
PS (Grade ≤ 1/Grade **≥** 2)	(45/51)	(193/0)	<0.0001***
Grade **≤** 1 (%)	47	100	
Grade **≥** 2 (%)	53	0	
Tumor type			
Superficial (%)	47	49	0.71
Exophytic (%)	42	40	0.80
Infiltrative (%)	11	11	1.0

***P < 0.001. Age is presented as the mean ± SD. PS = performance status

^198^Au grains were inserted under local anesthesia. The nominal activity of each seed was 185 MBq, and single plane implants were routinely performed. After implantation, the patients were cared for in a shielding room for 3–7 days until the total radiation activity decreased to a level of 700 MBq. Five radiotherapists took turns to perform the insertion of the ^198^Au grains. The radiation exposure to the operators was <40 µSv during each procedure. The most commonly prescribed dosage was 70 Gy over 7 days. The dosages were prescribed according to the dosage system reported by Paterson and Parker. Spacers were routinely prepared for brachytherapy performed after 1987 to prevent osteoradionecrosis. The grading system of postoperative complications defined by Shibuya *et al.* was used, as shown in Table 2 [2]. All of the treatments were completed without any problems.

**Table 2.** Definitions of soft tissue and mandibular complications

Grade	Soft tissue complication	Mandibular complication
**0**	No ulcer	No change
**I**	Transient ulcer disappeared within 6 months	Transient bone exposure disappeared spontaneously
**II**	Incurable ulcer lasted over 6 months	Bone necrosis healed by conservative treatment
**III**	Severe ulcer necessitating operation	Severe bone necrosis necessitating operation

We examined the outcomes in terms of cause-specific survival, overall survival and local control/recurrence rates. The survival curves were calculated using the Kaplan-Meier method. The outcomes in the different groups were compared using a log-rank test. Univariate and multivariate analyses were performed using the Cox proportional hazards model. Four-fold point correlation coefficients (known as φ coefficients) were evaluated for the correlation analysis of categorical variables. The Welch *t*-test and the Fisher exact test were used to compare differences in parametric continuous variables and categorical variables, respectively. All the analyses used the conventional *P* < 0.05 level of significance. The statistical analyses were performed using R, version 2.14.1.

## RESULTS

**Fig. 1.**
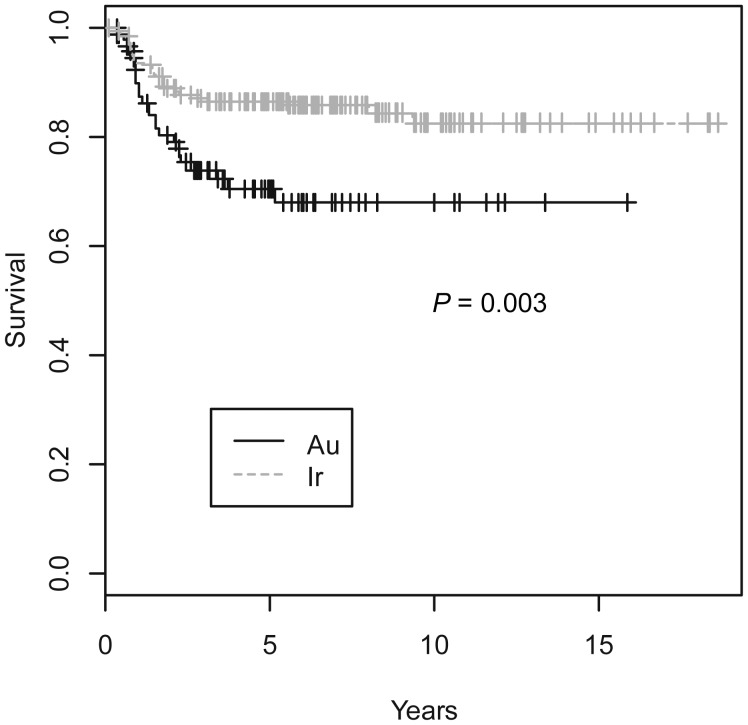
Cause-specific survival curves for the Au-group and the Ir-group.

**Fig. 2.**
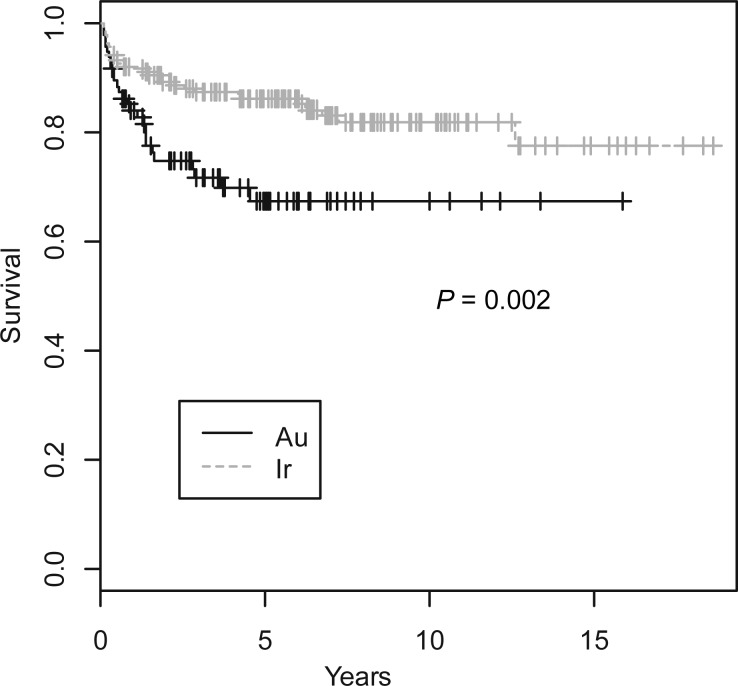
Local control survival curves for the Au-group and the Ir-group.

Table 3 compares the clinical outcomes of the two groups. The rate of postoperative complications, including tongue ulcers and osteoradionecrosis, were significantly lower in the Au-group than in the Ir-group. All the complications in the Au-group were Grade I, while about one-third of the complications in the Ir-group were Grade II. The 3-year and 5-year cause-specific survival rates were 73% and 71% in the Au-group, which were 15% and 16% lower than those in the Ir-group, respectively, which was statistically significant. Figure 1 shows the cause-specific survival rates. The 3-year and 5-year local control rates were 71% and 68% in the Au-group, which were 17% and 16% lower than those in the Ir-group, which again was statistically significant. Figure 2 shows the local control survival curves. The overall survival rates are also shown in Table 3.

**Table 3:** Clinical outcomes

	Au-group (*n* = 96)	Ir-group (*n* = 193)	*P*-value
Prescribed dose (Gy)	70 (8)	70 (0)	
Follow-up period (yrs)	3.9 ± 3.3	6.6 ± 4.2	
Deceased patients (tongue cancer)	2.0 ± 1.4	2.0 ± 2.2	
Deceased patients (other cause)	4.2 ± 5.2	5.0 ± 3.3	
Surviving patients	5.2 ± 3.0	7.7 ± 4.0	
Postoperative complications (%)	8.3	19	0.02*
Grade I (%)	100	69	
Grade II (%)	0	31	
Local recurrence (%)	28	16	0.03*
Cause-specific death (%)	26	15	0.02*
Lymph node metastases (%)	24	26	0.67
Cause-specific survival rate			0.003**
3 yrs (%)	73	88	
5 yrs (%)	71	87	
Overall survival rate			0.001**
3 yrs (%)	57	75	
5 yrs (%)	51	72	
Local control rate			0.002**
3 yrs (%)	71	88	
5 yrs (%)	68	86	

The follow-up period is presented as the mean ± SD. The prescribed dosage is presented as the median (interquartile range) because it did not have a normal distribution. *P < 0.05, **P < 0.01.

Table 4 shows the results of the univariate analyses. In the Au-group, infiltrative-type lesions caused significantly more lymph-node metastases and tended to cause more local recurrences and deaths, although the differences were not significant. Superficial-type lesions tended to cause fewer deaths, but the difference was not significant. The prescribed dose was inversely and significantly related to the rate of local recurrence (unadjusted hazard ratio = 0.94, *P* = 0.03). Women had significantly more lymph-node metastases than men and tended to have a higher mortality rate, although the difference was not significant. In the Ir-group, superficial-type lesions caused significantly fewer lymph node metastases and deaths.

**Table 4.** Univariate analysis

Au-group	Local recurrence		Cause-specific death		Lymph node metastasis	
	UHR	*P*-value	UHR	*P*-value	UHR	*P*-value
Sex (male vs female)	0.65	0.27	0.47	0.06	0.4	0.03*
Age	1.01	0.47	1.02	0.35	1.01	0.68
Tumor type (superficial)	0.74	0.51	0.43	0.09	0.60	0.32
Tumor type (infiltrative)	2.10	0.14	2.15	0.11	3.27	0.02*
T-factor (T2 vs T1)	1.98	0.11	1.15	0.72	1.34	0.49
Prescribed dose	0.94	0.03*	0.96	0.23	1.00	0.90
**Ir-group**						
Sex (male vs female)	1.07	0.86	1.39	0.45	0.95	0.85
Age	1.00	0.78	1.01	0.49	0.99	0.25
Tumor type (superficial)	0.57	0.17	0.23	0.005**	0.51	0.03*
Tumor type (infiltrative)	1.48	0.43	1.72	0.22	0.97	0.93
T-factor (T2 vs T1)	1.90	0.14	1.65	0.25	1.36	0.32
Prescribed dose	0.99	0.90	0.97	0.65	0.98	0.65

UHR = unadjusted hazard ratio. *P < 0.05, **P < 0.01.

Table 5 shows the results of the multivariate analyses. In the Au-group, infiltrative-type lesions caused significantly more lymph node metastases. Superficial-type lesions tended to cause fewer deaths, but the difference was not significant. The prescribed dose was inversely related to the local recurrence rate, but not significantly. Women tended to have more lymph-node metastases and cause-specific deaths, but the differences were not significant. In the Ir-group, superficial-type lesions caused significantly fewer lymph node metastases and deaths.

**Table 5.** Multivariate analysis

Au-group	Local recurrence		Cause-specific death		Lymph node metastasis	
	AHR	*P*-value	AHR	*P*-value	AHR	*P*-value
Sex (male vs female)	0.76	0.53	0.48	0.10	0.42	0.06
Age	1.01	0.68	1.01	0.55	1.00	0.92
Tumor type (superficial)	0.80	0.65	0.40	0.07	0.59	0.33
Tumor type (infiltrative)	1.62	0.37	2.14	0.14	3.58	0.02*
T-factor (T2 vs T1)	1.32	0.56	0.53	0.18	0.69	0.46
Prescribed dose	0.95	0.10	0.97	0.29	1.01	0.82
**Ir-group**						
Sex (male vs female)	1.08	0.85	1.35	0.50	0.92	0.79
Age	1.00	0.76	1.01	0.42	0.99	0.29
Tumor type (superficial)	0.62	0.27	0.22	0.005**	0.49	0.03*
Tumor type (infiltrative)	1.39	0.52	1.57	0.33	0.86	0.72
T-factor (T2 vs T1)	1.63	0.27	1.05	0.92	1.14	0.69
Prescribed dose	0.99	0.90	0.93	0.32	0.96	0.46

AHR = adjusted hazard ratio. *P < 0.05, **P < 0.01.

Correlation analyses showed that cause-specific deaths were related to local recurrences (φ = 0.49) and lymph-node metastases (φ = 0.49), but no correlation was seen between local recurrences and lymph-node metastases (φ = 0.097) in the Au-group. Similar results were also observed in the Ir-group (φ = 0.35, 0.35 and 0.10, respectively).

## DISCUSSION

With the rapid aging of Japan's population, the incidence rates of tongue cancer with advanced age are also rising [10]. Most of these patients cannot undergo invasive surgery, brachytherapy using ^192^Ir hairpins or ^197^Cs needles, or chemoradiation [7]. One of the curative treatment modalities left is ^198^Au grains, because these grains can be applied under local anesthesia without producing pain or discomfort [7]. Our results showed that the differences in the cause-specific survival and local control rates between the Au-group and the Ir-group were less than 17%.

Thirty years ago, Slanina *et al.* reported their experience using ^189^Au grains in 32 patients with Stage I–III tongue cancer [12]. However, the mean age of their subjects was 61 years, which was 10 years younger than that of our study. Furthermore, they did not report any comorbid diseases in their patients. The 5-year cause-specific survival rate of their patients was comparable with that of our patients.

Infiltrative-type lesions tended to cause more lymph-node metastases, while superficial-type lesions tended to cause fewer. These results were consistent with those of a former study reported by Kirita *et al.* [13]. Our data also showed a tendency for the macroscopic appearances of the tumors to affect the cause-specific survival rates in the two groups. These results were consistent with previous studies [14–16]. To clarify this relation, we performed correlation analyses, as mentioned in the *Results*. The results showed that lymph-node metastasis was related to the cause-specific survival rates, in agreement with former studies [14, 17–19]. This finding suggests that the macroscopic appearances of the tumors affect the cause-specific deaths through lymph-node metastasis.

The univariate analysis showed that women had significantly more lymph-node metastases than men. The multivariate analysis also showed a tendency for women to have more lymph-node metastases, but the difference was not significant. This result was inconsistent with Yoshida *et al.*'s study, which concluded that a male gender was a risk factor for lymph-node metastasis and cause-specific survival [20]. Further study of a larger population may be needed to clarify this inconsistency.

Our study did not show that age was a prognostic factor in either group. This result agrees with results of some previous studies [20–23]. However, this finding remains controversial because Yamazaki *et al.* showed that age was a potential prognostic factor [24].

## CONCLUSION

In conclusion, our study showed the clinical outcomes of brachytherapy using ^198^Au grains for early-tongue-cancer patients of advanced age or poor performance status. With the aging of Japan's population, this minimal invasive therapy is becoming increasingly important and could be used as a curative treatment for these patients.
